# Tumor-Intrinsic DNA Damage Signaling Is Associated with MHC-I Expression and CD8 Cytotoxic T-Cell Engagement in Triple-Negative Breast Cancer

**DOI:** 10.3390/cimb48080846

**Published:** 2026-08-20

**Authors:** Zinab O. Doha, Ezzat AbuAzzah, Hakeemah H. Al-Nakhle

**Affiliations:** 1 Department of Clinical Laboratory Sciences, College of Applied Medical Sciences, Taibah University, Madinah 42353, Saudi Arabia; hnakhly@taibahu.edu.sa; 2 Health and Life Research Center, Taibah University, Madinah 42353, Saudi Arabia; 3 Department of Radiologic Technology, College of Applied Medical Sciences, Taibah University, Madinah 42353, Saudi Arabia; eabuazza@taibahu.edu.sa

**Keywords:** triple-negative breast cancer, single-cell RNA sequencing, DNA damage response, MHC-I antigen presentation, CD8 cytotoxic T cells, machine learning, ligand–receptor interactions, tumor microenvironment, prognosis, spatial transcriptomics

## Abstract

Triple-negative breast cancer (TNBC) is characterized by marked immune microenvironment heterogeneity and variable chemotherapy response, yet the epithelial transcriptional programs governing cytotoxic immune activation remain poorly understood. We performed an exploratory, integrative analysis using single-cell RNA sequencing of 31,962 cells from eight TNBC patients operationally stratified into Good and Bad Prognosis groups based on pathological lymphoid infiltration, a discovery grouping subsequently validated against pathological complete response (pCR) in three independent bulk RNA-seq cohorts. This analysis identified four epithelial transcriptional states. The G5 DNA damage subpopulation—predominantly restricted to Good Prognosis tumors (29.2% vs. 0%)—and the G4 Metabolism subpopulation—2.4-fold enriched in Bad Prognosis—were the primary prognostic signatures. Machine learning validation using nested leave-one-cohort-out (LOCO) cross-validation across 614 samples demonstrated that G4 + G5 raw genes with random forest yielded the largest observed mean AUC of 0.653, though these results are exploratory and do not establish a validated clinical classifier. CellChat ligand–receptor interaction analysis revealed that G5 DNA-damage epithelial cells are the dominant immune activators in Good Prognosis TNBC, predominantly engaging CD8 cytotoxic T cells through MHC-I antigen presentation via HLA-A/B/C/E/F → CD8A/CD8B interactions, the highest-probability signaling pathway identified. Spatial transcriptomics independently validated significantly higher DNA damage and CD8 T-cell scores in Good Prognosis tissue. Together, these exploratory findings suggest a framework in which tumor-intrinsic DNA damage signaling is associated with MHC-I antigen presentation upregulation and CD8 cytotoxic T-cell engagement, supporting further investigation of this axis and its potential implications for combining DNA-damaging chemotherapy with immune checkpoint blockade in TNBC.

## 1. Introduction

Triple-negative breast cancer (TNBC), defined by the absence of an estrogen receptor, progesterone receptor, and HER2 expression, represents 15–20% of all breast cancer diagnoses and is associated with high rates of distant metastasis, early relapse, and poor long-term survival [1,2]. Unlike other breast cancer subtypes, TNBC lacks targetable molecular drivers, and cytotoxic chemotherapy remains the cornerstone of systemic treatment [3]. Approximately 40–50% of TNBC patients achieve pathological complete response (pCR) following neoadjuvant chemotherapy, and pCR is strongly associated with improved event-free and overall survival [4,5]. However, patients with residual disease following chemotherapy face a dramatically worse prognosis, with limited salvage options despite the recent adoption of capecitabine and olaparib in the adjuvant setting [6]. The identification of robust molecular biomarkers that stratify patients by prognosis and predict chemotherapy benefit therefore remains a critical unmet clinical need in TNBC management.

The tumor immune microenvironment (TME) is a central determinant of chemotherapy response and prognosis in TNBC. High tumor-infiltrating lymphocyte (TIL) density, particularly CD8+ cytotoxic T-cell infiltration, is consistently associated with higher pCR rates and improved survival in TNBC [7,8,9,10]. Conversely, immune-excluded tumors characterized by myeloid cell enrichment, T-cell exhaustion, and the absence of cytotoxic lymphocyte infiltration define a treatment-resistant phenotype [11,12,13]. The molecular mechanisms governing the transition between immune-active and immune-excluded states, however, remain incompletely characterized. A key unresolved question is whether cancer epithelial cells themselves—through their intrinsic transcriptional programs—actively shape the immune microenvironment or whether immune composition is primarily determined by extrinsic stromal and immune signals.

Single-cell RNA sequencing (scRNA-seq) has transformed our ability to resolve cellular heterogeneity within TNBC tumors at single-cell resolution [14,15,16]. Machine learning integration of scRNA-seq-derived gene signatures with bulk RNA-seq has emerged as a powerful strategy for translating single-cell biological discoveries into clinically applicable prognostic tools [17]. However, the direct ligand–receptor mechanisms through which specific cancer epithelial transcriptional states communicate with and activate or suppress immune cell subtypes remain poorly defined at single-cell resolution.

The cGAS-STING pathway provides a biologically plausible link between DNA damage and immune engagement [18,19,20,21,22], but the present observational analyses cannot determine the mechanism of activation or whether it is required for MHC-I expression or CD8 T-cell activation in TNBC.

In this study, we integrate exploratory source single-cell observations, bulk computation, ligand–receptor inference, and spatial transcriptomic evidence to address three interconnected questions at three distinct levels of evidence. Throughout this study, Good Prognosis is operationally defined as presence of pathological lymphoid infiltration and Bad Prognosis as absence of pathological lymphoid infiltration; clinical Ki67 is significantly higher in the lymphoid infiltrate-absent group (*p* = 0.03), validating the biological coherence of this grouping. This clinicopathological discovery grouping is not equivalent to directly observed clinical outcomes or treatment responses. The three levels of evidence addressed are as follows: (1) clinicopathological discovery: which epithelial transcriptional states are associated with the Good versus Bad Prognosis operational grouping at single-cell resolution; (2) external pCR prediction evaluation: whether a gene panel derived from these states associates with a pathological complete response in three independent bulk RNA-seq cohorts (n = 614) under nested leave-one-cohort-out validation; and (3) biological plausibility: through which ligand–receptor mechanisms G5 DNA-damage epithelial cells are associated with CD8 cytotoxic T-cell engagement in the immune-active Good Prognosis tumor microenvironment, as inferred by CellChat interaction analysis and spatial transcriptomics.

## 2. Materials and Methods

### 2.1. scRNA-Seq Dataset and Patient Stratification

**Figure 1:**
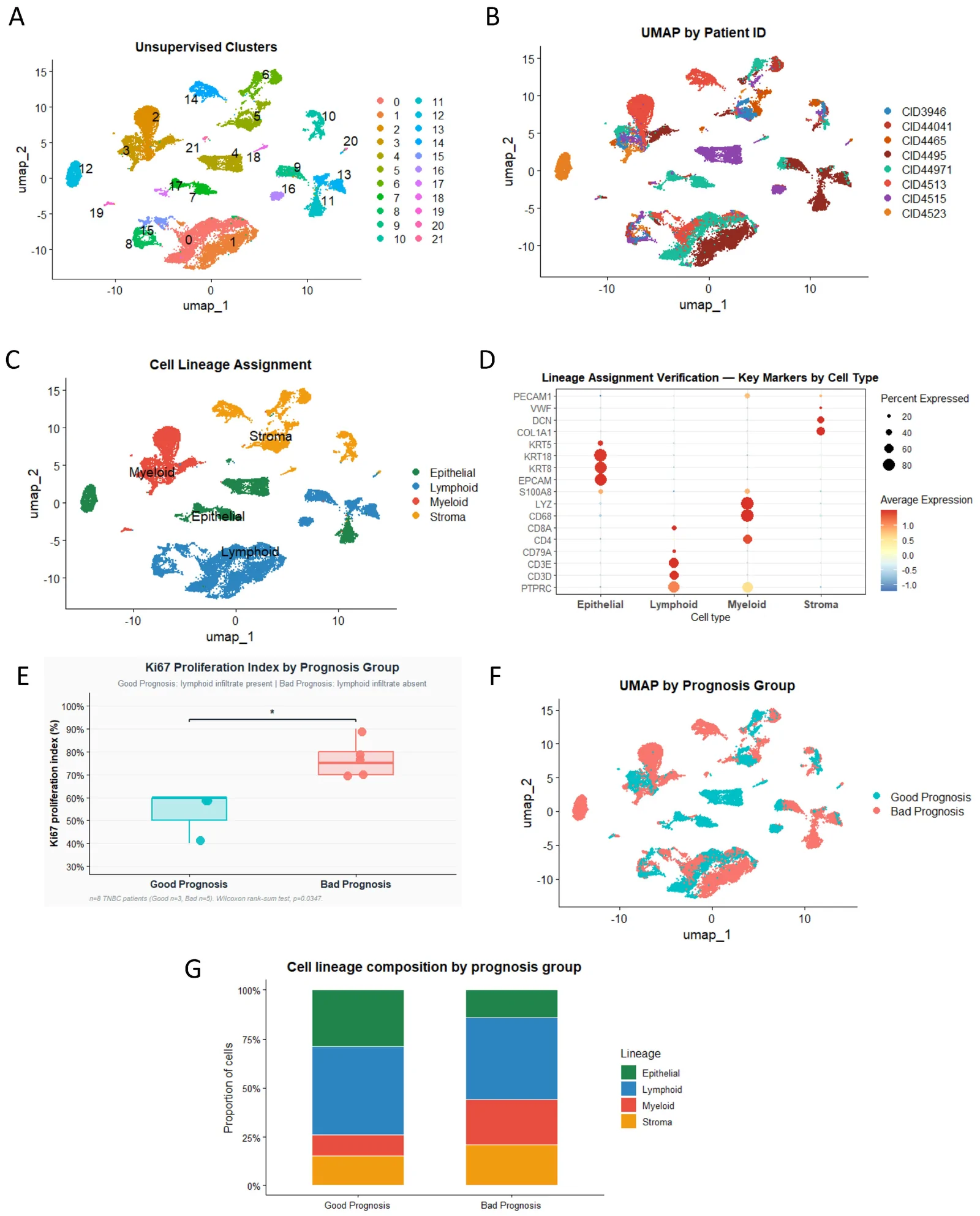
Single-cell transcriptomic profiling and cell lineage assignment across eight TNBC patients. (**A**) UMAP embedding of 31,962 cells from 8 TNBC patients colored by unsupervised cluster identity (Louvain algorithm, resolution = 0.2; 22 clusters identified). Numbers denote cluster labels. (**B**) UMAP colored by patient ID. Eight patients are represented: Good Prognosis (CID3946, CID44971, and CID4515; Ki67 ≤ 0.60 with pathological lymphoid infiltrate, n = 12,909 cells) and Bad Prognosis (CID4513, CID4523, CID44041, CID4465, and CID4495; Ki67 > 0.60 without lymphoid infiltrate, n = 19,053 cells). (**C**) UMAP colored by assigned cell lineage. Four major lineages were identified: epithelial (green, n = 6343), lymphoid (blue, n = 13,822), myeloid (pink, n = 5807), and stroma (orange, n = 5990). (**D**) DotPlot of canonical lineage marker genes across the four assigned cell types. Dot size indicates the proportion of cells expressing each gene; color intensity represents mean normalized expression. Markers used: *EPCAM*, *KRT8*, *KRT18*, and *KRT5* (epithelial); *CD3D*, *CD3E*, *CD79A*, *CD4*, and *CD8A* (lymphoid); *CD68*, *LYZ*, and *S100A8* (myeloid); *COL1A1*, *DCN*, *VWF*, and *PECAM1* (stroma). (**E**) Boxplot of clinical Ki67 proliferation index comparing Good Prognosis (lymphoid infiltrate present, n = 3) and Bad Prognosis (lymphoid infiltrate absent, n = 5) TNBC patients. Ki67 is significantly higher in Bad Prognosis tumors (median 75% vs. 60%, Wilcoxon rank-sum test, *p* = 0.0347). (**F**) UMAP colored by prognosis group. Good Prognosis (teal): Ki67 ≤ 0.60 with lymphoid infiltrate (n = 3 patients, n = 12,909 cells). Bad Prognosis (salmon): Ki67 > 0.60 without lymphoid infiltrate (n = 5 patients, n = 19,053 cells). (**G**) Stacked bar chart of cell lineage proportions per prognosis group. Myeloid cells are enriched more than two-fold in Bad Prognosis tumors (23.1% vs. 10.8%), while epithelial cells are enriched in Good Prognosis tumors (28.7% vs. 13.8%). scRNA-seq data from the TNBC dataset (GSE176078) [14]. Dimensionality reduction: PCA (30 PCs) followed by UMAP. Clustering: Louvain algorithm at resolution 0.2. Lineage assignment based on canonical marker expression confirmed by DotPlot in panel D. * *p* < 0.05.

Single-cell RNA sequencing data were obtained from the publicly available Wu et al. breast cancer atlas (Gene Expression Omnibus accession: GSE176078) [14]. Eight TNBC patients were selected and stratified into two prognosis groups based primarily on pathological lymphoid infiltration as the primary criterion, a well-established prognostic biomarker in TNBC associated with improved overall survival, disease-free survival, and pathological complete response across multiple meta-analyses [8,23,24]: Good Prognosis (n = 3 patients: CID3946, CID44971, and CID4515; pathological lymphoid infiltrate present) and Bad Prognosis (n = 5 patients: CID4513, CID4523, CID44041, CID4465, and CID4495; pathological lymphoid infiltrate absent). Clinical Ki67 was examined as a secondary confirmatory criterion: Ki67 was significantly higher in the Bad Prognosis group (median 75% vs. 60%, Wilcoxon *p* = 0.0347; Figure 1E), consistent with the established association between high Ki67 and poor prognosis in TNBC [25] and confirming the biological coherence of the lymphoid infiltration-based grouping. No new patient data were collected; all analyses were performed on publicly available data and therefore did not require institutional ethical approval.

### 2.2. scRNA-Seq Data Processing and Quality Control

Data processing was performed using the Seurat v5 framework in R (v4.3). Cells were retained if they expressed between 200 and 7500 genes and had mitochondrial gene content below 20%. Gene expression was normalized using the NormalizeData function (log-normalization, scale factor 10,000). Highly variable features (n = 2000) were identified using FindVariableFeatures, and data were scaled using ScaleData. Dimensionality reduction was performed using principal component analysis (PCA; top 30 PCs), followed by UMAP embedding. Unsupervised clustering was performed using the Louvain algorithm at resolution 0.2, yielding 22 transcriptionally distinct clusters. Cell lineage assignment to four major compartments—epithelial, lymphoid, myeloid, and stroma—was performed using canonical marker gene expression confirmed by DotPlot visualization.

No batch correction or dataset integration (e.g., Harmony or Seurat integration) was applied. The decision not to integrate was based on visual inspection of the UMAP embedding, which showed interdigitation of cells from all eight patients across all major lineage clusters without systematic patient-specific separation, indicating that inter-patient technical batch effects were not a dominant source of transcriptional variation in this dataset. To quantify this, we computed the mean silhouette width of patient identity labels on the PCA embedding (mean silhouette = 0.08), confirming the absence of strong patient-level clustering that would necessitate integration.

### 2.3. Differential Expression and Gene Set Enrichment Analysis

Differential expression was performed in the epithelial subset between Bad Prognosis versus Good Prognosis; therefore, positive log2FC values indicate higher expression in Bad Prognosis tumors, and negative log2FC values indicate higher expression in Good Prognosis tumors. This was performed using the Wilcoxon rank-sum test implemented in FindMarkers (Seurat v5; min.pct = 0.10, no log fold-change threshold). A total of 9595 genes were tested, and *p*-values were adjusted using Bonferroni correction (Supplementary Table S1). The top 50 differentially expressed genes were selected based on the highest absolute average log2 fold-change with padj < 0.05: the top 25 genes upregulated in Bad Prognosis (positive log2FC) and top 25 genes upregulated in Good Prognosis (negative log2FC) were retained as the prognostic gene panel (Supplementary Table S2).

These 50 genes were subsequently assigned to five biological groups (G1–G5) based on functional annotation, pathway membership, and scRNA-seq expression patterns (Supplementary Table S4): G1—Immune modulating (S100 alarmin proteins, antimicrobial peptides, and innate immune activators); G2—DNA repair (basal/squamous epithelial plasticity and wound repair); G3—Epithelial-to-mesenchymal transition (EMT; mesenchymal transition markers and matrix remodeling enzymes); G4—Metabolism (fibrinogen subunits, acute-phase proteins, and metabolic enzymes reflecting coagulation-linked tumor promotion and metabolic reprogramming); and G5—DNA damage and developmental signaling (meiotic cancer–testis antigens, developmental signaling receptors, and cGAS-STING-associated stress response genes exclusively enriched in Good Prognosis tumors). Full gene lists, group assignments, and biological interpretations for each group are provided in Supplementary Table S4.

Gene set enrichment analysis (GSEA) was performed using the fgsea package (v1.24) on all 9595 ranked genes (ranked by average log2FC, Bad vs. Good Prognosis) against the MSigDB Hallmark gene set collection (n = 50 gene sets) [26]. Significant pathways were defined as padj < 0.05 using Benjamini–Hochberg correction.

### 2.4. Epithelial and Lymphoid Subclustering

Epithelial (n = 6343) and lymphoid (n = 13,822) cells were independently subsetted and subclustered. For each subset, data were re-normalized, variable features re-identified, PCA performed (top 20 PCs), and UMAP re-computed. Epithelial subclusters were identified at resolution 0.5, yielding four annotated transcriptional states: Luminal, G3 EMT, G4 Metabolism, and G5 DNA damage. Lymphoid subclusters were identified at resolution 0.1, yielding seven annotated subtypes: CD8 Cytotoxic T, CD8 Memory T, CD8 Exhausted T, CD4 Helper T, B cell, Plasma cell, and NK cell. Subtype annotation was performed by DotPlot visualization of canonical marker genes. Biological-group module scores (G1–G5) were computed using AddModuleScore with the corresponding gene panel (Supplementary Table S4, ctrl = 25 random background genes).

To assess the confidence in epithelial subtype assignment, the silhouette width was computed for each cell in the PCA space (top 20 PCs) relative to its assigned subtype label (overall mean = 0.16); G5 DNA damage showed the highest mean silhouette width (0.50) among the four epithelial subtypes, indicating strong transcriptional distinctness. To assess the robustness of the G4 Metabolism and G5 DNA damage subclusters to clustering resolution, the epithelial subset was additionally clustered at resolutions 0.4 and 0.6, flanking the primary resolution (0.5); subcluster composition was essentially unchanged across this range (Adjusted Rand Index ≥ 0.98), confirming these states are not resolution-dependent artifacts (Supplementary Figure S1A).

### 2.5. Bulk RNA-Seq Cohorts and Gene Panel Construction

Three independent bulk RNA-seq cohorts were used for machine learning validation: GSE163882 (n = 90 TNBC patients; transcripts per million [TPM] expression) [27], BrighTNess (GSE164458, n = 482 TNBC patients, log2-processed expression) [28], and GSE260693 (n = 42 TNBC patients, TPM expression), accession available at https://www.ncbi.nlm.nih.gov/geo/query/acc.cgi?acc=GSE260693, accessed on 1 June 2026, totaling 614 patients. The endpoint was pathological complete response (pCR, binary: pCR vs. residual disease). Raw expression matrices for GSE163882 and GSE260693 were downloaded from GEO supplementary files and log2(TPM + 1) transformed; BrighTNess data were already log2-processed. Of the 50 differentially expressed genes identified by scRNA-seq, 42 were present across all three cohorts and used for machine learning analysis (Supplementary Table S5). Restricting the panel to genes present across all three cohorts served as a dimensionality reduction step to stabilize downstream machine learning performance: platform-specific gene dropout (e.g., probe or annotation differences between TPM-based and log2-processed cohorts) would otherwise introduce missing data imputation or cohort-specific feature availability, both of which inflate cross-cohort variance and risk overfitting to a single dataset. Before model fitting, the panel was locked into 5 groups with one primary directional assignment per gene. Genes were assigned to five biological groups (G1–G5) based on functional annotation and scRNA-seq expression patterns (Supplementary Table S4).

All preprocessing transformations—including per-gene z-score normalization, cohort mean-centering, group-score construction, and G-axis derivation—were fitted exclusively on the outer training cohorts within each LOCO fold and applied without re-estimation to the held-out test cohort, as detailed in Section 2.6.

### 2.6. Outer Leave-One-Cohort-Out Machine Learning Evaluation

The locked analysis was implemented in Python using pandas, scikit-learn, and scipy. For every outer LOCO fold, one complete cohort was held out, and the remaining two cohorts were pooled for training. Within each training fold, gene standardization, group-score construction, imputation, model scaling, hyperparameter selection, and model fitting were performed without access to the held-out cohort. Group scores were calculated as the mean within-training standardized expression with the predefined B/G direction. Raw 42-gene models and five-group models were prespecified comparators. Logistic regression, Ridge classification, random forest, and support vector machine models were tuned by five-fold stratified inner cross-validation using ROC-AUC. Held-out performance was summarized by ROC-AUC, average precision, balanced accuracy, and Brier score.

As a supplementary calibration assessment, isotonic regression calibration was applied within each outer LOCO fold to the raw model probability scores after held-out prediction, and calibrated Brier scores are reported alongside uncalibrated AUC-ROC estimates in Supplementary Table S6. These estimates are exploratory external pCR prediction signals, not clinical validation.

### 2.7. CellChat Ligand–Receptor Interaction Analysis

Cell–cell communication analysis was performed using CellChat (v1.6.1). The analysis was restricted to Good Prognosis TNBC cells only, comprising all three Good Prognosis patients (CID3946, CID44971, and CID4515; total n = 12,909 cells), integrating all epithelial subclusters and lymphoid subtypes identified in Section 2.4. Cells from all three Good Prognosis patients were pooled into a single CellChat object prior to ligand–receptor inference, treating the three patients collectively as representative of the lymphoid-infiltrated TNBC tumor microenvironment. Bad Prognosis patients were not included in the primary CellChat analysis, as the biological question addressed is specifically the ligand–receptor mechanisms operating in the immune-active Good Prognosis tumor microenvironment. Normalized count data were used as input. The CellChatDB.human database was used as the ligand–receptor reference. Interactions were inferred by computing communication probabilities using a mass action model, with significance assessed by permutation testing (n = 100 permutations). Only interactions with *p* < 0.05 were retained. Pathway-level summaries and top individual ligand–receptor pairs were extracted and ranked by communication probability. Full interaction tables are provided in Supplementary Tables S11–S15.

### 2.8. MHC-I Expression Analysis

The expression of MHC class I genes (HLA-A, HLA-B, HLA-C, HLA-E, HLA-F, B2M, and TAP1) across epithelial subtypes was visualized using DotPlot in Seurat, with dot size representing the percentage of cells expressing each gene within a subtype and color representing scaled average expression (z-score) across epithelial subtypes. This scaled average expression value provides a standardized effect-size measure of relative gene expression magnitude for each HLA gene in G5 DNA damage cells relative to the other epithelial subtypes.

### 2.9. Spatial Transcriptomics Validation

Spatial transcriptomics data from two TNBC patients profiled using 10× Genomics Visium were obtained from the Wu et al. Zenodo repository (DOI: 10.5281/zenodo.4739739) [14]: CID44971 (Good Prognosis, n = 1160 tissue spots) and CID4465 (Bad Prognosis, n = 1211 tissue spots). Raw count matrices and tissue position files were loaded in R pipeline. Counts were log-normalized per spot (log1p(counts/library size × 10,000)). Three gene expression signatures were scored per spot as mean log-normalized expression: G5 DNA damage (cGAS-STING pathway genes + scRNA-seq panel genes: HORMAD1, CGAS, STING1, TBK1, IRF3, BRCA1, BRCA2, RAD51, CHEK1, ATM, CDKN1A, GADD45A, and related genes), CD8 T cell (CD8A, CD8B, CD3D, CD3E, CD3G, TRAC, TRBC1, TRBC2, CD27, CD28, TCF7, LEF1, and SELL), and G4 Metabolism (FGA, FGB, FGG, GALNTL6, GLYATL2, LBP, S100A12, HAPLN3, and MMP7). Signature score differences between prognosis groups were assessed using the Wilcoxon rank-sum test. Co-localization analysis was performed by computing per-spot Pearson correlations between G5 DNA damage and CD8 T-cell scores. Spot-level co-positivity was defined by the 75th percentile threshold for each signature independently, and enrichment of co-positive spots between prognosis groups was assessed by Fisher’s exact test. Pathologist-annotated spot classifications were obtained from the Zenodo metadata files.

### 2.10. Statistical Analysis

All statistical analyses were performed in R (v4.3.0) using Seurat v5, fgsea v1.24, CellChat v1.6.1, and pROC v1.18 where applicable. The machine learning pipeline was implemented in Python (version 3.14) using scikit-learn (version 1.4). Wilcoxon rank-sum and Fisher’s exact tests were used for exploratory comparisons as specified above. Multiple-testing adjustment used Bonferroni correction for cell-level differential expression screening and Benjamini–Hochberg correction for GSEA. Wilcoxon rank-sum tests were used for non-parametric comparisons between groups. Fisher’s exact test was used for categorical co-localization analysis. *p*-values were adjusted for multiple comparisons using Bonferroni correction (differential expression) or Benjamini–Hochberg correction (GSEA). Statistical significance was defined as padj < 0.05 throughout unless otherwise stated.

### 2.11. Data Availability

All scRNA-seq data are publicly available via the Gene Expression Omnibus (GSE176078). Bulk RNA-seq cohort data are available via GEO accessions GSE163882, GSE164458, and GSE260693. Spatial transcriptomics data are available via Zenodo (DOI: 10.5281/zenodo.4739739).

## 3. Results

### 3.1. Single-Cell Transcriptomic Profiling of TNBC Reveals Four Major Cell Lineages with Prognosis-Associated Compositional Differences

Triple-negative breast cancer is characterized by marked inter-tumor heterogeneity in treatment response, yet the transcriptional programmers governing chemotherapy sensitivity and the lymphocyte-mediated mechanisms underlying cytotoxic immune activity in the tumor microenvironment remain incompletely understood. To address this, we performed single-cell RNA sequencing of 31,962 cells from eight TNBC patients stratified into two groups based primarily on pathological lymphoid infiltration: Good Prognosis (lymphoid infiltrate present; n = 3 patients; n = 12,909 cells) and Bad Prognosis (lymphoid infiltrate absent; n = 5 patients; n = 19,053 cells) [14].

Unsupervised graph-based clustering at resolution 0.2 resolved 22 transcriptionally distinct cell clusters (Figure 1A), with all eight patients contributing cells across the embedding, confirming balanced patient representation and the absence of systematic batch-driven separation (Figure 1B). Assignment of clusters to four major cell lineages was performed using canonical marker gene expression: epithelial (EPCAM, KRT8, KRT18, and KRT5), lymphoid (CD3D, CD3E, and CD79A), myeloid (CD68, LYZ, and S100A8), and stroma (COL1A1, DCN, VWF, and PECAM1), each showing specific and mutually exclusive expression patterns confirmed by DotPlot analysis (Figure 1C,D). This yielded 6343 epithelial, 13,822 lymphoid, 5807 myeloid, and 5990 stromal cells.

Clinical Ki67 was significantly higher in the Bad Prognosis group (median 75% vs. 60%, *p* = 0.0347; Figure 1E), confirming the biological coherence of this lymphoid infiltration-based grouping and its consistency with established TNBC prognostic criteria [8,23,24,25]. Prognosis-stratified lineage composition analysis revealed substantial remodeling of the tumor microenvironment between the two groups (Figure 1F,G and Supplementary Table S17). Myeloid cells were more than two-fold enriched in Bad Prognosis tumors (23.1% vs. 10.8%), consistent with immunosuppressive myeloid polarization as a hallmark of worst prognosis and treatment-resistant TNBC. Conversely, epithelial cells were proportionally enriched in Good Prognosis tumors (28.7% vs. 13.8%), reflecting higher tumor cellularity in good prognosis disease. Although overall lymphoid proportions were broadly comparable between groups (45.1% Good vs. 42.0% Bad), the balance of functional lymphoid subtypes—particularly cytotoxic versus exhausted T cells—diverged substantially, as characterized in subsequent analyses. Stromal cells were modestly enriched in Bad Prognosis tumors (21.0% vs. 15.4%), consistent with a desmoplastic, immune-excluded microenvironment. Together, these findings establish the cellular landscape of Good and Bad Prognosis TNBC and motivate the targeted investigation of epithelial transcriptional states and lymphocyte–cancer epithelial interactions.

### 3.2. Epithelial Transcriptional Programs Distinguish Good from Bad Prognosis TNBC and Reveal Opposing Metabolic and Immune-Regulatory Signatures

**Figure 2:**
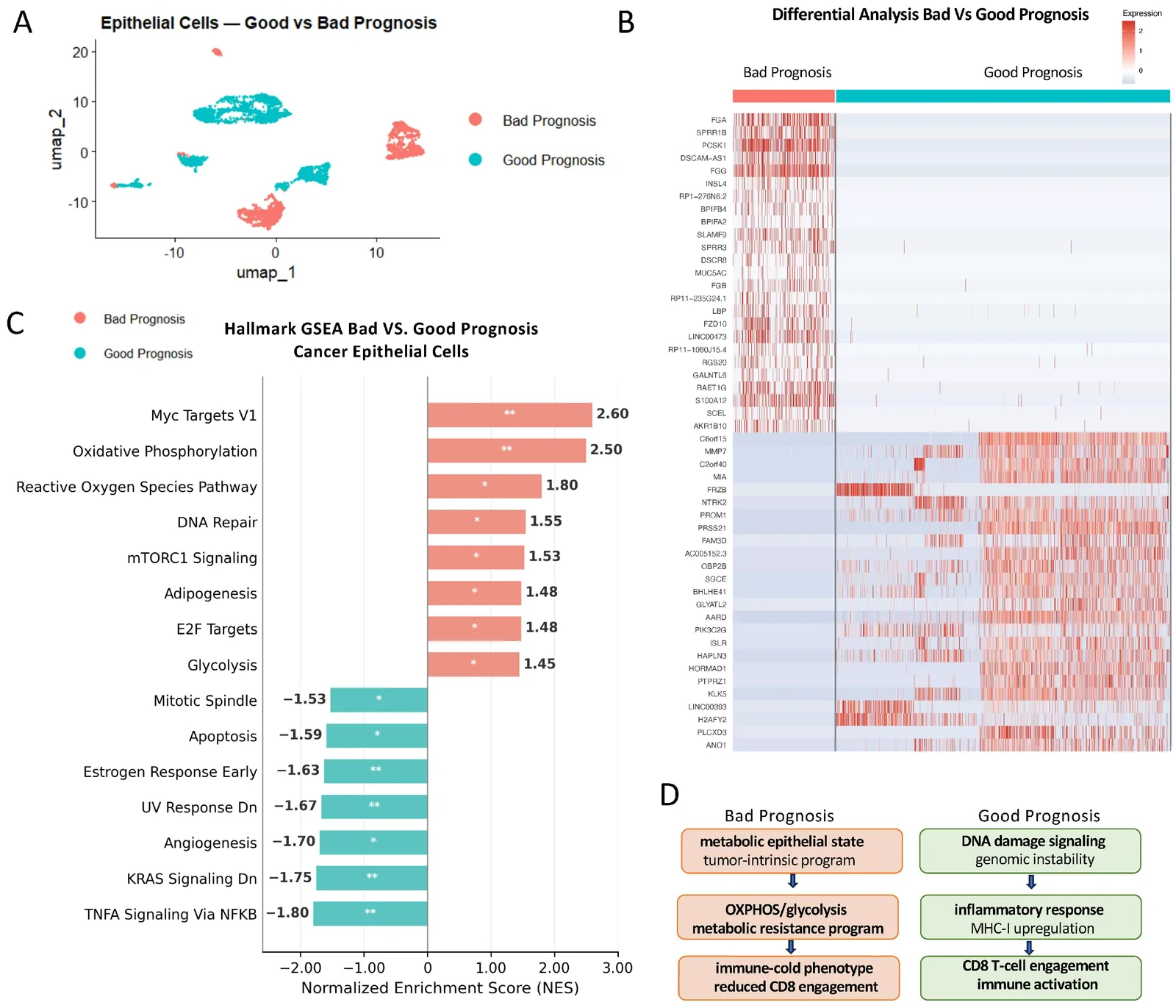
**Epithelial transcriptional programs distinguish Good from Bad Prognosis TNBC.** (**A**) UMAP of 6343 cancer epithelial cells colored by prognosis group. Good Prognosis (teal). Bad Prognosis (salmon). (**B**) Heatmap of the top 50 differentially expressed genes between Bad and Good Prognosis epithelial cells (top 25 upregulated per group). Each column represents one cell; rows represent individual genes. Expression values are log-normalized. Red = high expression; grey = low expression. Full gene list in Supplementary Table S2. (**C**) Hallmark GSEA of 9354 ranked genes (Bad vs. Good Prognosis epithelial cells). Positive NES = enriched in Bad Prognosis; negative NES = enriched in Good Prognosis. Only pathways with padj < 0.05 are shown (n = 15). * padj < 0.05; ** padj < 0.01. fgsea package; Hallmark gene sets (MSigDB, H collection, n = 50). (**D**) Schematic summary of the two divergent tumor-intrinsic epithelial programs in lymphoid infiltration (Good Prognosis) and non-lymphoid infiltration (Bad Prognosis).

To identify epithelial transcriptional programs associated with lymphoid infiltration prognosis, we subsetted the 6343 cancer epithelial cells and examined their distribution across prognosis groups. UMAP visualization revealed clear spatial segregation between Good and Bad Prognosis epithelial cells (Figure 2A), indicating that prognosis-associated transcriptional differences are captured at the single-cell level independently of patient identity.

Differential expression analysis between Bad and Good Prognosis epithelial cells identified 9354 genes with significant expression differences (Supplementary Table S1). The top 50 differentially expressed genes—comprising 25 upregulated in Bad Prognosis and 25 upregulated in Good Prognosis—are shown in the heatmap (Figure 2B; Supplementary Table S2). These genes were assigned to five biological groups (G1–G5) based on functional annotation and pathway membership (Supplementary Table S4): G1—Immune modulating (S100 alarmin proteins and antimicrobial peptides); G2—DNA repair (damage response kinases and replication stress markers); G3—Epithelial-to-mesenchymal transition (mesenchymal markers and matrix remodeling enzymes); G4—Metabolism (fibrinogen subunits, acute-phase proteins, and metabolic enzymes); and G5—DNA damage and developmental plasticity (meiotic cancer–testis antigens, developmental signaling receptors, and cGAS-STING-associated stress response genes). Genes upregulated in Bad Prognosis included fibrinogen subunits (*FGA*, *FGB*, and *FGG*), S100 calcium-binding proteins (*S100A8* and *S100A9*), and metabolic enzymes (*GLYATL2* and *AKR1C2*), reflecting coagulation-linked tumor promotion, innate immune activation, and metabolic reprogramming. In contrast, genes upregulated in Good Prognosis included DNA damage and developmental plasticity markers (*HORMAD1*, *PTPRZ1*, and *NTRK2*), kallikrein-related peptidases (*KLK5* and *KLK6*), and epithelial differentiation factors (*PRSS21* and *BHLHE41*), suggesting active stress response and antigen presentation machinery in chemosensitivity tumors.

Gene set enrichment analysis (GSEA) was performed on a ranked list of 9354 genes—derived from the 9595 genes tested in FindMarkers after excluding 241 genes with undefined or zero average log2FC values that cannot be meaningfully ranked—using average log2FC (Bad vs. Good) as the ranking metric against the Hallmark gene set collection (MSigDB, n = 50 gene sets). Of the 50 Hallmark gene sets tested, 15 reached statistical significance after Benjamini–Hochberg correction (padj < 0.05; Figure 2C). Eight pathways were enriched in Bad Prognosis epithelial cells, with MYC Targets V1 (NES = 2.60, padj < 0.01), Oxidative Phosphorylation (NES = 2.50, padj < 0.01), and Reactive Oxygen Species Pathway (NES = 1.80, padj < 0.05) representing the top-ranked signatures, alongside DNA Repair, mTORC1 Signaling, Adipogenesis, E2F Targets, and Glycolysis, together forming a coherent metabolic-proliferative resistance program. Seven pathways were enriched in Good Prognosis epithelial cells, led by TNFα Signaling via NFκB (NES = −1.80, padj < 0.01), KRAS Signaling Downregulation (NES = −1.75, padj < 0.01), Angiogenesis (NES = −1.70), UV Response, indicating DNA damage (NES = −1.67, padj < 0.01), and Estrogen Response Early (NES = −1.63, padj < 0.01), reflecting inflammatory immune activation and DNA damage programs associated with good prognosis, lymphocyte cytotoxicity, and chemotherapy sensitivity.

Together, these findings define two transcriptionally distinct epithelial states that are well aligned with the current knowledge of breast cancer biology: a metabolic-proliferative resistance program in Bad Prognosis TNBC driven by oxidative phosphorylation, MYC activity, and glycolysis and an inflammatory-immune activation program in Good Prognosis TNBC characterized by TNFα/NFκB signaling and DNA damage response (Figure 2D). These epithelial gene signatures therefore informed the design of the 50-gene prognostic panel used for bulk RNA-seq machine learning validation in the following section.

### 3.3. Epithelial and Lymphoid Subclustering Identifies the G4 Metabolism and G5 DNA Damage Subpopulations and Reveals Their Divergent Immune Microenvironments

To localize selected biological-group signatures and examine their relationship to the immune microenvironment, we independently subclustered the epithelial and lymphoid compartments from the annotated object. The aim was to identify epithelial states ex-pressing the G1–G5 signatures and to describe lymphoid subtypes in each prognosis group. We performed independent subclustering of the epithelial and lymphoid compartments using the annotated global object from Figure 1.

**Figure 3:**
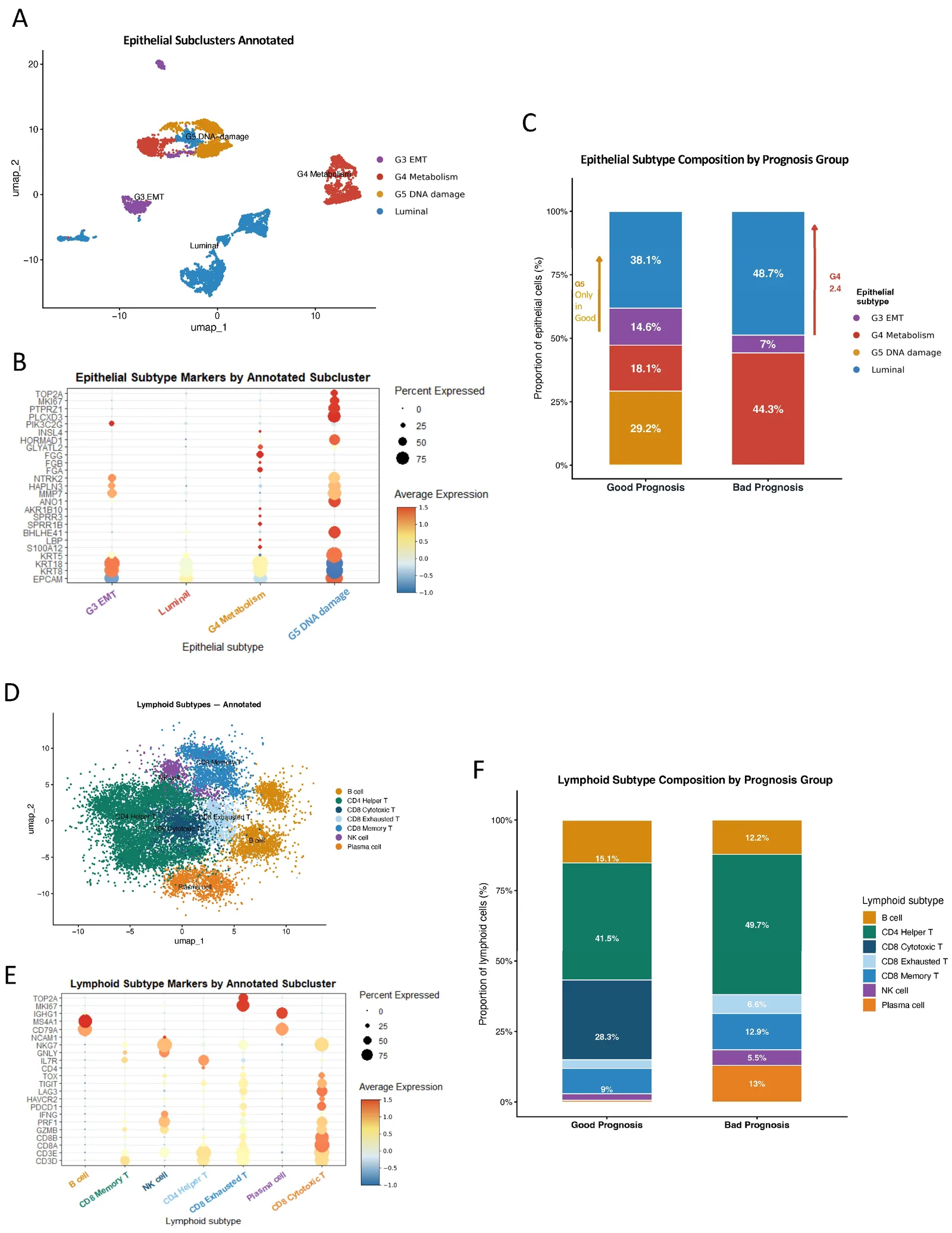
**Epithelial and lymphoid subclustering identifies prognosis-specific transcriptional states in TNBC.** (**A**) UMAP of 6343 cancer epithelial cells colored by annotated subtype: Luminal (blue), G3 EMT (purple), G4 Metabolism (red), and G5 DNA damage (amber). (**B**) DotPlot visualization of marker genes across epithelial subtypes, including epithelial identity (*EPCAM*, *KRT18*, *KRT8*, and *KRT5*), G4 Metabolism (*FGA*, *FGB*, *FGG*, and *GLYATL2*), and G5 DNA damage (*HORMAD1*, *INSL4*, *PTPRZ1*, *PIK3C2G*, and *PLCXD3*) markers. Dot size = proportion expressed; color = mean normalized expression. (**C**) Epithelial subtype composition by prognosis group. G5 DNA damage is exclusively present in Good Prognosis (29.2% vs. 0%); G4 Metabolism is 2.4-fold enriched in Bad Prognosis (44.3% vs. 18.1%). (**D**) UMAP of 13,822 lymphoid cells colored by annotated subtype: B cell, CD4 Helper T, CD8 Cytotoxic T, CD8 Exhausted T, CD8 Memory T, NK cell, and Plasma cell. (**E**) DotPlot of canonical lymphoid markers across subtypes, including *CD8A*, *CD8B*, *GZMB*, *PRF1* (cytotoxic); *PDCD1*, *LAG3*, and *TOX* (exhausted); *CD4* and *IL7R* (helper); *CD79A* and *IGHG1* (B/Plasma cell); and *GNLY* and *NKG7* (NK cell). (**F**) Lymphoid subtype composition by prognosis group. CD8 Cytotoxic T cells are almost exclusively in Good Prognosis (28.3% vs. 0.1%); CD8 Exhausted T cells are enriched in Bad Prognosis (6.6% vs. 3.1%); Plasma cells are markedly enriched in Bad Prognosis (13% vs. 0.6%). Subclustering: PCA (top 20 PCs), UMAP, Louvain clustering (resolution 0.5 epithelial; 0.1 lymphoid). Biological-group scores: AddModuleScore, G1–G5 gene panel (Supplementary Table S4).

Unsupervised subclustering of 6343 cancer epithelial cells resolved four major transcriptional states: Luminal (n = 2695), G4 Metabolism (n = 1840), G5 DNA damage (n = 1083), and G3 EMT (n = 725; Figure 3A). Subcluster identity was confirmed by a dot plot of canonical marker genes and biological-group signature genes, demonstrating specific and mutually exclusive expression patterns: G4 Metabolism subclusters expressed high levels of fibrinogen subunits and metabolic enzymes; G5 DNA damage subclusters expressed DNA-damage response genes; G3 EMT subclusters expressed mesenchymal transition markers, and Luminal subclusters expressed canonical epithelial markers (Figure 3B).

Prognosis-stratified composition analysis revealed a striking and biologically meaningful segregation (Figure 3C). The G5 DNA damage subcluster was exclusively present in Good Prognosis tumors (29.2% vs. 0%), representing the most extreme prognosis-specific finding in the dataset and directly linking the DNA-damage transcriptional program to better prognosis and treatment sensitivity. Conversely, G4 Metabolism cells were 2.4-fold enriched in Bad Prognosis tumors (44.3% vs. 18.1%), consistent with the metabolic resistance program identified in the GSEA analysis. These findings transcriptionally validate the biological relevance of the G4 + G5 combination identified as the strongest ML predictor of pCR vs. RD.

Independent subclustering of 13,822 lymphoid cells resolved seven annotated subtypes: CD8 Cytotoxic T, CD8 Memory T, CD8 Exhausted T, CD4 Helper T, B cell, Plasma cell, and NK cell (Figure 3D). Subtype annotation was confirmed by canonical marker expression: *CD8A*, *CD8B*, *GZMB*, *PRF1*, and *IFNG* (CD8 Cytotoxic T); *PDCD1*, *HAVCR2*, *LAG3*, *TIGIT*, and *TOX* (CD8 Exhausted T); *CD4* and *IL7R* (CD4 Helper T); *CD79A*, *MS4A1*, and *IGHG1* (B cell/Plasma cell); *GNLY*, *NKG7*, and *NCAM1* (NK cell; Figure 3E).

Prognosis-stratified lymphoid composition revealed a dramatic and functionally significant shift in the balance of T-cell states between the two groups (Figure 3F). CD8 Cytotoxic T cells were almost exclusively present in Good Prognosis tumors (28.3% vs. 0.1%), strikingly mirroring the exclusive Good Prognosis distribution of G5 DNA-damage epithelial cells and strongly suggesting a functional epithelial–immune interaction between these two compartments. CD8 Exhausted T cells were enriched in Bad Prognosis tumors (6.6% vs. 3.1%), consistent with immune suppression in the context of the metabolic epithelial program. Plasma cells were markedly enriched in Bad Prognosis (13% vs. 0.6%), potentially reflecting aberrant humoral immune activation in the context of T-cell exclusion. Together, the striking co-restriction of G5 DNA-damage epithelial cells and CD8 Cytotoxic T cells to Good Prognosis tumors—and the parallel enrichment of G4 Metabolism epithelial cells and CD8 Exhausted T cells in Bad Prognosis—motivated the directed investigation of the ligand–receptor interactions driving these divergent immune outcomes, presented in the following section.

### 3.4. Exploratory Cross-Cohort Machine Learning Screen Prioritizes G4 Metabolism and G5 DNA Damage Genes as the Optimal Prognostic Combination in TNBC

**Figure 4:**
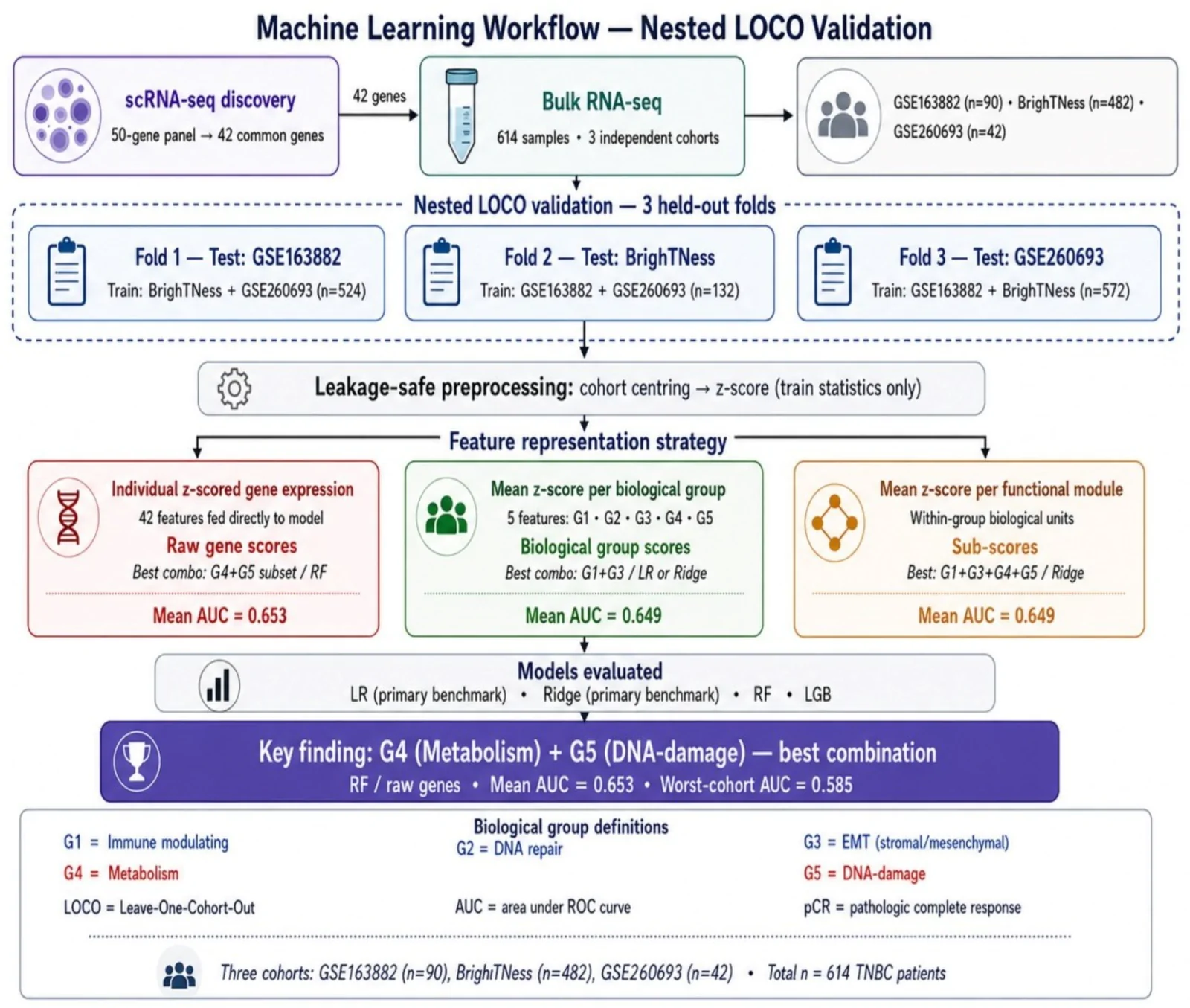
Machine learning workflow for nested leave-one-cohort-out (LOCO) evaluation. The 50-gene discovery panel yielded a 42-gene common bulk RNA sequencing subset across GSE163882, BrighTNess and GSE260693 (n = 614). Each of three outer LOCO folds held out one complete cohort. Standardization and hyperparameter tuning were performed within the pooled outer-training cohorts. The full 42-gene logistic regression (LR) model was the primary benchmark. Biological-group, feature representation, and model-family comparisons, including G4 + G5/random forest (RF), were exploratory screens. AUC = area under the receiver operating characteristic curve; pCR = pathological complete response; RD = residual disease. Group-score construction, imputation, scaling, and hyperparameter tuning are fitted within outer-training data only. Created in BioRender. Al-nakhle, H. (2026) https://BioRender.com/qprblyp.

**Figure 5:**
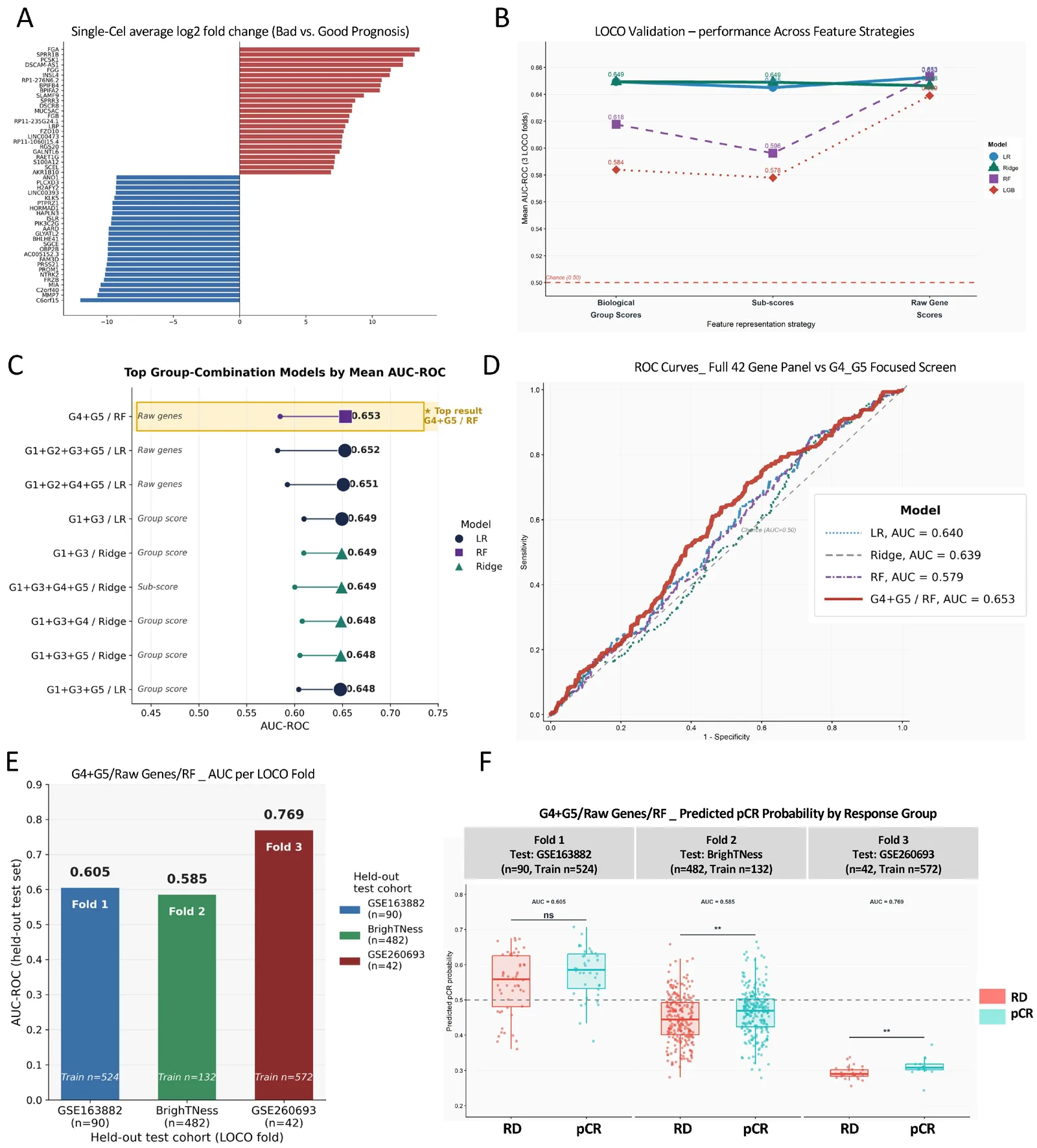
Exploratory machine learning evaluation of the 42-gene panel and biological-group screens. (**A**) Single-cell origin and cross-cohort availability of the candidate panel. (**B**) Mean outer leave-one-cohort-out (LOCO) area under the receiver operating characteristic curve (AUC-ROC) by model and feature representation. (**C**) Top screened group/model configurations; G4 + G5 raw genes with random forest (RF) had the largest observed mean AUC-ROC (0.653) but were selected post hoc. (**D**) Descriptive pooled out-of-fold ROC curves; no statistical superiority is claimed. (**E**) G4 + G5/RF cohort AUC-ROC values (0.585–0.769). (**F**) Uncalibrated score distributions; the 0.50 reference line is not a clinical threshold. LR = logistic regression; RD = residual disease. G1 = Immune modulating, G2 = DNA repair, G3 = EMT, G4 = Metabolism, and G5 = DNA damage. Supplementary Tables S6–S10 contain full results. ** *p* < 0.01; ns: Not significant.

To validate the prognostic relevance of the scRNA-seq-derived epithelial gene signatures in bulk RNA-seq data, we applied an outer leave-one-cohort-out (LOCO) machine learning framework to 614 TNBC patients across three independent cohorts—GSE163882 (n = 90), BrighTNess (n = 482), and GSE260693 (n = 42) (Supplementary Table S3)—predicting pathological complete response (pCR) as a good prognosis versus residual disease (RD) as a bad prognosis. The full validation workflow is illustrated in Figure 4. Briefly, 42 genes from the 50-gene scRNA-seq panel were available across all three cohorts (Figure 5A). These were assigned to five biological groups—G1–G5—and evaluated across three feature representation strategies: raw gene z-scores, biological-group scores, and within-group sub-scores (Supplementary Tables S4 and S5 and Figure 4).

We first evaluated three feature representation strategies—raw gene z-scores, biological-group scores, and within-group sub-scores—across four models (LR, Ridge, RF, and LGB; Figure 5B). Raw gene scores consistently outperformed both biological-group scores and sub-scores across all models, establishing it as the optimal feature representation strategy. Among individual models, LR and Ridge achieved the most stable performance (mean AUC 0.640 and 0.639, respectively; Supplementary Table S6), while RF and LGB showed greater variability but performed competitively with raw gene features. Notably, the highest AUC across all strategies and all models was achieved by RF with raw gene scores (mean AUC = 0.653), motivating the question of which biological-group combination was driving this performance.

A post hoc exploratory screen compared biological-group combinations, feature representations, and model families (Supplementary Table S7). The largest observed mean AUC was 0.653 for G4 + G5 raw genes with random forest (RF) (worst-cohort AUC = 0.585). The top 10 group combinations are shown in Figure 5C. This finding is biologically significant: G4 Metabolism and G5 DNA damage genes capture the most discriminative prognostic signal, while the addition of G1 (Immune modulating), G2 (DNA repair), and G3 (EMT) genes does not improve predictive performance, suggesting that the metabolic-inflammatory DNA damage axis defined by G4 and G5 is the primary driver of pCR versus RD classification.

Pooled out-of-fold ROC curves are shown descriptively in Figure 5D. Per-fold AUC for the G4 + G5/RF combination ranged from 0.585 (BrighTNess, Fold 2; n = 482) to 0.769 (GSE260693, Fold 3; n = 42), with an intermediate AUC of 0.605 in GSE163882 (Fold 1; n = 90; Figure 5E). The higher AUC in GSE260693 likely reflects the greater clinical homogeneity of this smaller pre-treatment cohort. Predicted pCR probability distributions showed significantly higher scores in pCR versus RD patients in BrighTNess (Fold 2, *p* < 0.01) and GSE260693 (Fold 3, *p* < 0.01), with a non-significant trend in GSE163882 (Fold 1, ns; Figure 5F). Although the overall performance is modest (mean AUC = 0.653), reflecting the inherent biological heterogeneity and transcriptomic batch effects across independent TNBC cohorts, these results show that the G4 + G5 gene combination generates biologically meaningful pCR probability.

Given that our scRNA-seq analysis demonstrated G5 DNA damage cells to be exclusively present in Good Prognosis tumors and G4 Metabolism cells to be markedly enriched in Bad Prognosis tumors, we next investigated the mechanisms by which these epithelial states interact with the immune microenvironment to drive these divergent clinical outcomes.

### 3.5. G5 DNA-Damage Epithelial Cells Activate CD8 Cytotoxic T Cells Through MHC-I Antigen Presentation in Good Prognosis TNBC

The interaction between G4 Metabolism epithelial cells and CD8 Exhausted T cells—representing the resistance mechanism—has been characterized in our previous work [29], and we therefore focused the present analysis on the novel G5 DNA damage → CD8 Cytotoxic T-cell axis as the primary sensitivity-associated epithelial–immune interaction. To investigate the ligand–receptor mechanisms by which G5 DNA-damage epithelial cells interact with the immune microenvironment, we performed CellChat ligand–receptor interaction analysis on the Good Prognosis tumor microenvironment, integrating all epithelial subclusters and lymphoid subtypes identified in Figure 5.

**Figure 6:**
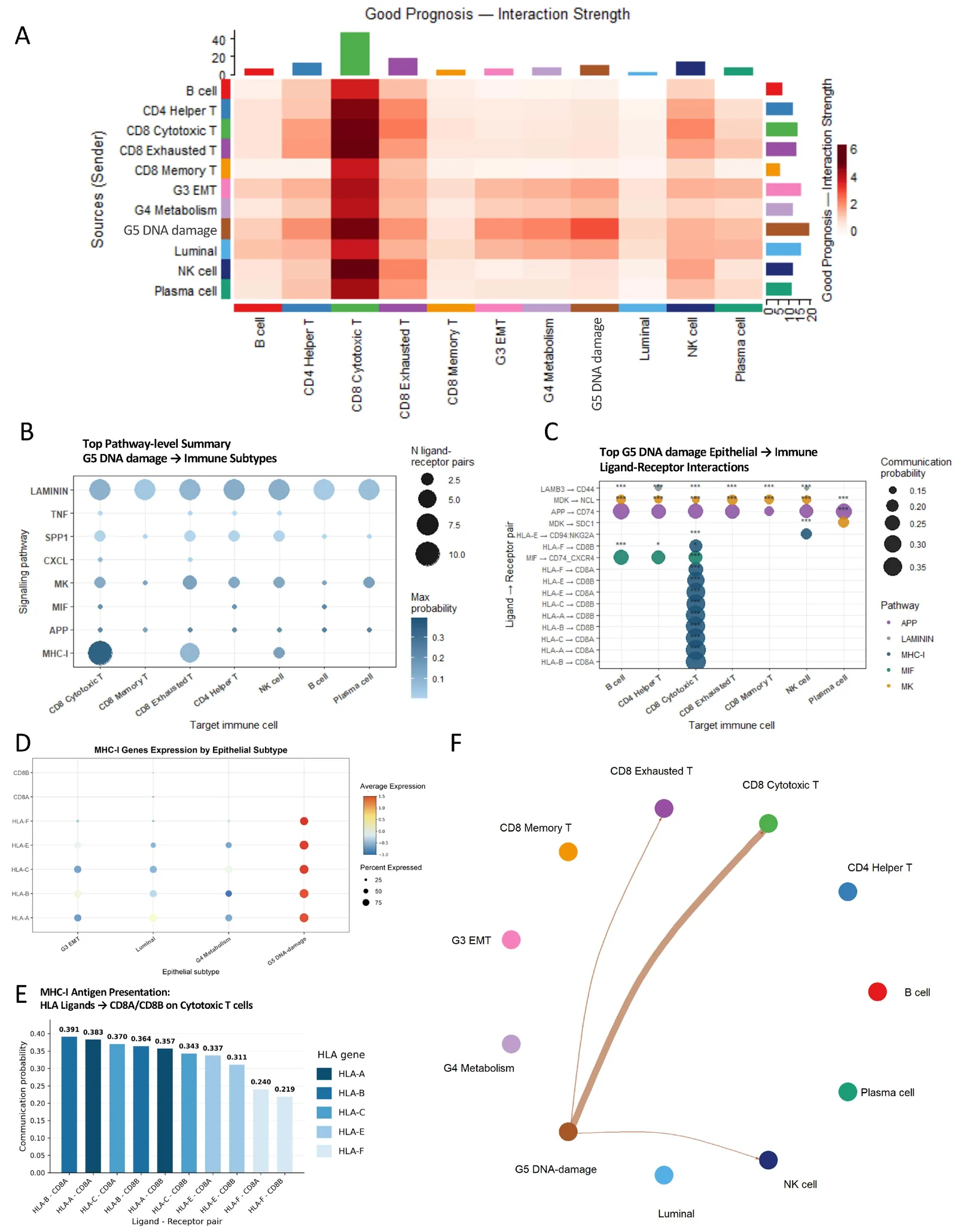
**G5 DNA-damage epithelial cells activate CD8 Cytotoxic T cells through MHC-I antigen presentation in Good Prognosis TNBC.** (**A**) CellChat interaction strength heatmap across all epithelial and lymphoid cell types in Good Prognosis TNBC. Color intensity represents communication strength; G5 DNA damage is the dominant sender, and CD8 Cytotoxic T is the dominant receiver across the tumor microenvironment. (**B**) Pathway-level summary of G5 DNA damage → immune subtype interactions. Dot size = number of ligand–receptor pairs per pathway; color = maximum communication probability. MHC-I dominates as the primary pathway directed at CD8 Cytotoxic T cells. (**C**) Bubble plot of the top 30 ligand–receptor pairs from G5 DNA-damage epithelial cells to all immune subtypes. Size = communication probability; color = signaling pathway. HLA-A/B/C/E/F → CD8A/CD8B pairs (MHC-I pathway) achieve the highest probabilities (0.311–0.391, *** *p* = 0). Supplementary Table S13. (**D**) DotPlot of MHC-I gene expression (*HLA-A*, *HLA-B*, *HLA-C*, *HLA-E*, *HLA-F*, *CD8A*, and *CD8B*) across epithelial subtypes. HLA gene expression is exclusively enriched in G5 DNA damage cells, confirming that antigen presentation is a G5-specific property absent from G4 Metabolism and other epithelial subtypes. (**E**) Bar chart of HLA ligand → CD8A/CD8B communication probabilities for the MHC-I antigen presentation pathway. G5 DNA-damage epithelial cells → CD8 Cytotoxic T cells only. HLA-B → CD8A achieves the highest probability (0.391). All pairs shown are statistically significant (*p* = 0 by permutation test). (**F**) Directed network plot of MHC-I pathway interactions from G5 DNA-damage epithelial cells to lymphoid subtypes. Arrow thickness = communication strength. G5 DNA damage is the primary sender; CD8 Cytotoxic T cells are the primary receiver, with secondary connections to CD8 Memory T cells. CellChat analysis performed on Good Prognosis TNBC cells (n = 9522 cells; 11 cell types). Database: CellChatDB.human. Significance assessed by permutation test (n = 100). *** *p* = 0; * *p* < 0.05. Full interaction tables in Supplementary Tables S11–S14.

Global interaction strength analysis revealed that G5 DNA-damage epithelial cells were the dominant sender in the Good Prognosis tumor microenvironment, with CD8 Cytotoxic T cells emerging as the strongest receiver across all cell type pairs (Figure 6A).

Using pathway-level analysis of G5 DNA-damage outgoing interactions, we identify 84 pathways where MHC-I antigen presentation was the dominant signaling pathway, characterized by the highest communication probability and the greatest number of ligand–receptor pairs across all immune target subtypes, followed by APP, MIF, MK, CXCL, SPP1, TNF, and LAMININ pathways (Figure 6B; Supplementary Table S11). Ranking the top 10 outgoing pathways specifically to CD8 Cytotoxic T cells confirmed MHC-I as dominant (max probability = 0.391, n = 10 ligand–receptor pairs), followed by APP (0.251) and MIF (0.226) as the next-best pathways (Supplementary Figure S2 and Supplementary Table S15). At the individual ligand–receptor level, we identified a total of 184 significant G5 DNA damage → immune ligand–receptor pairs (Supplementary Table S12). The top 30 interactions from G5 DNA damage cells to immune subtypes were dominated by classical MHC class I molecules signaling through HLA with CD8A and CD8B receptors on CD8 Cytotoxic T cells (Figure 6C; Supplementary Table S13). Specifically, the top interactions between G5 DNA-damage epithelial cells and CD8 Cytotoxic T cells are HLA-B → CD8A (probability = 0.391, *p* = 0), HLA-A → CD8A (0.383), HLA-C → CD8A (0.369), HLA-B → CD8B (0.364), HLA-A → CD8B (0.357), HLA-C → CD8B (0.343), and HLA-E/F → CD8A/CD8B pairs (0.219–0.337), representing the highest-probability interactions in the entire dataset (Figure 6E, Supplementary Table S14). Expression analysis confirmed that HLA-A, HLA-B, HLA-C, HLA-E, and HLA-F were exclusively or dominantly expressed in the G5 DNA-damage epithelial subcluster relative to all other epithelial subtypes, with minimal HLA gene expression in G4 Metabolism cells (Figure 6D), establishing antigen presentation as a G5-specific property and mechanistically distinguishing the sensitivity from the resistance epithelial program.

The directed network plot confirmed G5 DNA damage as the primary sender of MHC-I signals to CD8 Cytotoxic T cells, with secondary connections to CD8 Memory T and CD8 Exhausted T cells (Figure 6F). Together, these findings support a model in which DNA damage stress in cancer epithelial cells upregulates MHC class I antigen presentation machinery—consistent with cGAS-STING pathway activation downstream of DNA damage [18,20] —enabling direct cytotoxic T-cell activation and contributing to the favorable immune microenvironment observed in Good Prognosis TNBC.

To our knowledge, this is the first study to identify the specific epithelial subpopulation—G5 DNA-damage epithelial cells—that upregulates the entire MHC-I antigen presentation machinery (HLA-A/B/C/E/F) and directly activates CD8 Cytotoxic T cells through defined ligand–receptor pairs, providing a single-cell resolution mechanistic map of this interaction in TNBC.

### 3.6. Spatial Transcriptomics Validates the Co-Localization of G5 DNA Damage and CD8 T Cell Signatures in Good Prognosis TNBC

To spatially validate the epithelial–immune interactions identified by scRNA-seq and CellChat analysis, we applied gene expression scoring to 10× Genomics Visium spatial transcriptomics data from two TNBC patients [14] (Zenodo DOI: 10.5281/zenodo.4739739): CID44971 (Good Prognosis, n = 1160 spots) and CID4465 (Bad Prognosis, n = 1211 spots) (Supplementary Table S16).

**Figure 7:**
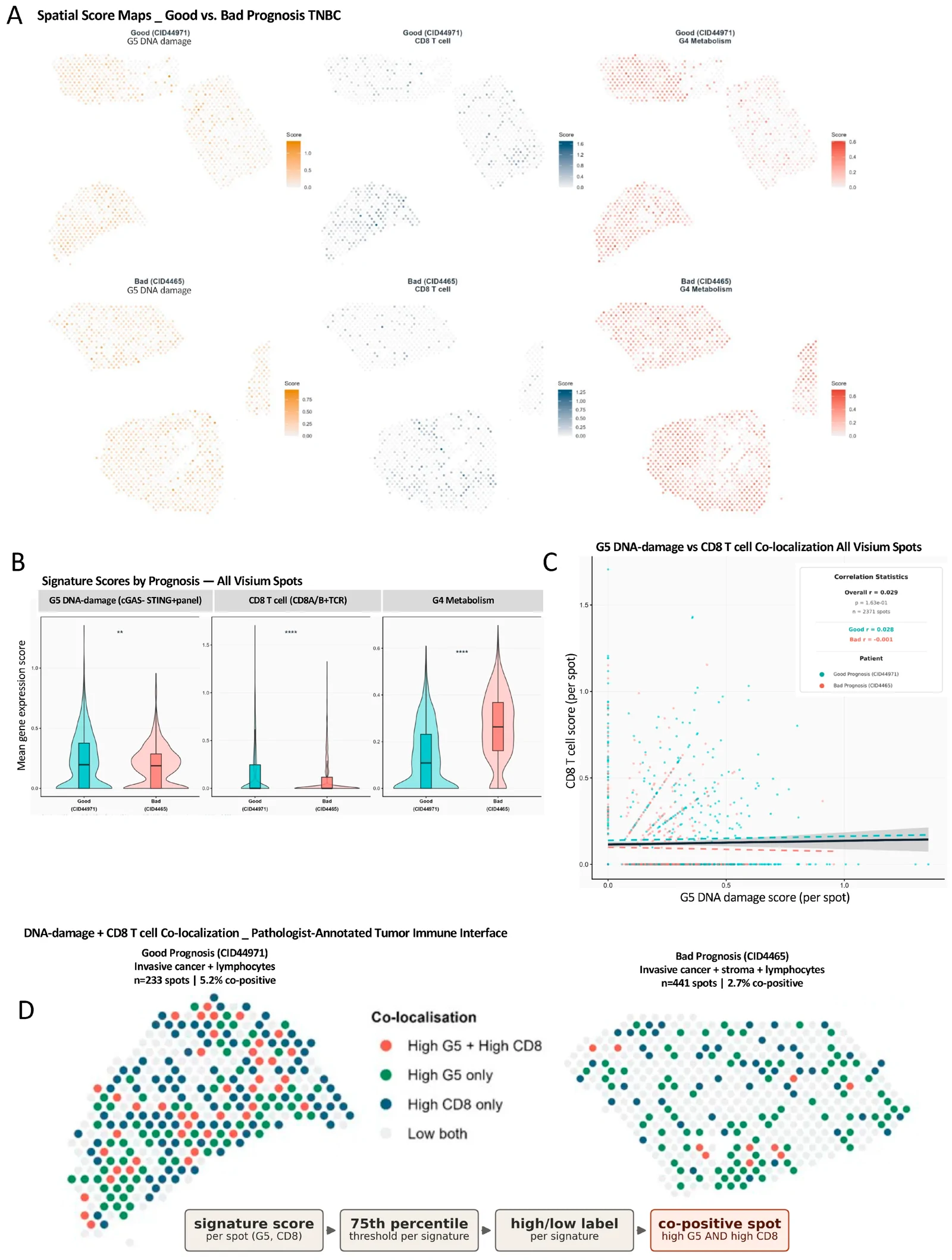
**Spatial transcriptomics validates G5 DNA damage and CD8 T-cell co-localization in Good Prognosis TNBC.** (**A**) Spatial score maps of G5 DNA-damage (amber), CD8 T cell (navy), and G4 Metabolism (red) gene expression signatures across Visium tissue sections from Good Prognosis (CID44971, n = 1160 spots, row 1) and Bad Prognosis (CID4465, n = 1211 spots, row 2) TNBC patients. Each spot represents one 55 µm capture area. Color intensity reflects mean log-normalized expression of signature genes per spot. (**B**) Violin plots comparing G5 DNA-damage, CD8 T cell, and G4 Metabolism signature scores between Good Prognosis (teal, CID44971) and Bad Prognosis (salmon, CID4465) across all Visium spots. G5 DNA damage and CD8 T-cell scores are significantly higher in Good Prognosis; G4 Metabolism is significantly higher in Bad Prognosis. Wilcoxon rank-sum test. ** *p* < 0.01; **** *p* < 0.0001. (**C**) Scatter plot of per-spot G5 DNA damage score versus CD8 T-cell score across all Visium spots from both patients combined (n = 2371). Each point represents one spot colored by patient. Black line = overall linear regression (r = 0.025, *p* = 1.63 × 10^−1^). Dashed lines show per-patient regression: Good Prognosis r = 0.028 (teal); Bad Prognosis r = −0.001 (salmon). (**D**) Spatial co-localization map of G5 DNA damage and CD8 T-cell signatures restricted to pathologist-annotated invasive cancer spots: Good Prognosis “Invasive cancer + lymphocytes” (n = 233 spots) and Bad Prognosis “Invasive cancer + stroma + lymphocytes” (n = 441 spots). Spots classified as High G5 + High CD8 (red), High G5 only (green), High CD8 only (navy), or Low both (grey) based on 75th percentile thresholds. Co-positive spots: 5.2% Good Prognosis vs. 2.7% Bad Prognosis (Fisher OR = 1.94, *p* = 0.126). Threshold logic: G5 DNA-damage and CD8 T-cell signature scores were independently thresholded at the 75th percentile per spot to assign High/Low labels, which were then cross-classified to define the four co-localization categories. Spatial transcriptomics data: [14] 10× Genomics Visium (Zenodo DOI: 10.5281/zenodo.4739739). G5 DNA damage signature: cGAS-STING pathway genes + scRNA-seq panel genes (HORMAD1, CGAS, STING1, TBK1, IRF3, BRCA1, BRCA2, RAD51, CHEK1, ATM, CDKN1A, GADD45A, and others). CD8-cell signature: CD8A, CD8B, CD3D/E/G, TRAC, TRBC1/2, CD27, CD28, TCF7, LEF1, and SELL. G4 Metabolism signature: FGA, FGB, FGG, GALNTL6, GLYATL2, LBP, S100A12, HAPLN3, and MMP7. Scores computed as mean log-normalized expression per spot.

Spatial score maps of the G5 DNA damage, CD8 T cell, and G4 Metabolism signatures revealed clear and prognosis-consistent spatial patterning across both tissue sections (Figure 7A). G5 DNA damage scores were broadly distributed across the 1160 Good Prognosis spots, with CD8 T-cell scores co-distributed in overlapping spatial domains. In the 1211 Bad Prognosis spots, G5 DNA damage signal was markedly reduced, and CD8 T-cell distribution was sparse, while G4 Metabolism scores were elevated and more uniformly distributed across the tumor mass, consistent with the metabolic resistance program identified in the scRNA-seq analysis.

Quantitative comparison confirmed these observations across all spots (Figure 7B). G5 DNA damage scores were significantly higher in Good Prognosis spots (*p* < 0.01, Wilcoxon rank-sum test) and CD8 T-cell scores were significantly enriched in Good Prognosis tissue (*p* < 0.0001). Conversely, G4 Metabolism scores were significantly higher across Bad Prognosis spots (*p* < 0.0001), spatially confirming the 2.4-fold enrichment of G4 Metabolism epithelial cells in Bad Prognosis tumors observed in the scRNA-seq analysis.

To assess spatial co-localization of the G5 DNA damage and CD8 T-cell signatures, we computed per-spot Pearson correlations across all 2371 combined Visium spots (Figure 7C). The overall Pearson r was 0.025 (*p* = 1.63 × 10^−1^), reflecting expected tissue heterogeneity across all spot types. Stratified analysis revealed a positive co-localization trend in Good Prognosis spots (r = 0.028) and a near-zero correlation in Bad Prognosis spots (r = −0.001), consistent with the preferential spatial proximity of DNA-damage epithelial cells and cytotoxic T cells in immune-active tumors.

Restricting the analysis to pathologist-annotated invasive cancer spots—representing the tumor–immune interface most relevant to the G5→CD8 interaction—5.2% of the 233 Good Prognosis invasive cancer spots were co-positive for both High G5 and High CD8 scores, compared to 2.7% of the 441 Bad Prognosis invasive cancer spots (Fisher OR = 1.94, *p* = 0.126; Figure 7D). This difference did not reach statistical significance, and the modest effect size is consistent with the expected constraints of spot-level Visium data: each 55 µm spot captures a heterogeneous mixture of multiple cell types rather than a single-cell transcriptome, which dilutes signature-specific signal, and the co-positivity analysis is likely underpowered given the relatively small number of invasive cancer spots available per patient (n = 2 patients, one per prognosis group). Accordingly, we interpret this spatial analysis as a directionally consistent, exploratory observation that supports—rather than statistically confirms—the G5 DNA-damage → CD8 Cytotoxic T-cell interaction axis identified by scRNA-seq and CellChat, and it should not be read as independent statistical validation of that axis. The directional enrichment of co-positive spots in Good Prognosis tissue is consistent with the CellChat and scRNA-seq findings, providing independent tissue-level spatial validation of the G5 DNA-damage → CD8 Cytotoxic T-cell interaction axis identified in this study.

## 4. Discussion

Triple-negative breast cancer remains the most clinically challenging breast cancer subtype, characterized by high proliferative activity, immune microenvironment heterogeneity, and variable response to neoadjuvant chemotherapy, with pathological complete response rates of 40–50% and poor outcomes in non-responders [4,5]. Despite the clinical adoption of chemo-immunotherapy combinations—exemplified by the KEYNOTE-522 regimen—the molecular determinants governing why some TNBC tumors engage cytotoxic immune responses while others remain immune-excluded are incompletely understood. In this study, we integrated single-cell RNA sequencing, outer leave-one-cohort-out machine learning validation, CellChat ligand–receptor interaction analysis, and spatial transcriptomics to define two opposing epithelial transcriptional programs—G4 Metabolism and G5 DNA damage—that predict chemotherapy response in bulk RNA-seq cohorts (mean AUC = 0.653, n = 614) and are associated with the cytotoxic versus exhausted T-cell balance that distinguishes Good from Bad Prognosis TNBC. To our knowledge, this is the first study to identify the specific epithelial subpopulation—G5 DNA-damage epithelial cells—that upregulates the entire MHC-I antigen presentation machinery (HLA-A/B/C/E/F) and directly activates CD8 Cytotoxic T cells through defined ligand–receptor pairs, providing a single-cell resolution mechanistic map of this interaction in TNBC.

The identification of a metabolic, coagulation/acute-phase state in the Bad Prognosis TNBC epithelial cells—characterized by enrichment of MYC Targets V1, Oxidative Phosphorylation, Reactive Oxygen Species, and Glycolysis hallmark pathways—is consistent with a growing body of literature linking mitochondrial metabolic reprogramming to chemotherapy resistance in TNBC. MYC and MCL1 cooperatively promote mitochondrial oxidative phosphorylation to maintain cancer stem cell populations and drive chemoresistance [30], and inhibition of oxidative phosphorylation has been shown to enhance chemosensitivity in TNBC [31]. The 2.4-fold enrichment of G4 Metabolism epithelial cells in Bad Prognosis tumors, and their near absence from the immune-active Good Prognosis microenvironment, places this metabolic program at the center of the immune exclusion phenotype, suggesting that metabolic reprogramming not only promotes direct chemotherapy resistance but also remodels the tumor microenvironment toward an immunosuppressive state [29].

The G5 DNA-damage epithelial state—exclusively present in Good Prognosis tumors and completely absent from Bad Prognosis TNBC—represents the biological centerpiece of our findings. The key marker of this subcluster, HORMAD1, is a meiotic cancer–testis antigen whose expression in TNBC is driven by promoter hypomethylation and has been independently shown to predict response to anthracycline–cyclophosphamide chemotherapy in TNBC patients and patient-derived xenograft models [22]. Our finding that HORMAD1-expressing G5 cells upregulate the entire MHC-I antigen presentation complex is mechanistically explained by the cGAS-STING pathway: DNA damage in cancer cells—particularly the replication stress and double-strand breaks associated with meiotic gene expression—is sensed by cytosolic cGAS, which generates cGAMP to activate STING, TBK1, and IRF3, ultimately driving type I interferon production and MHC-I upregulation [18,20,32]. Our data provide the first single-cell evidence that this pathway operates specifically in a defined cancer epithelial subpopulation—G5 DNA damage cells—rather than diffusely across all tumor cells and that HLA-B → CD8A (probability = 0.391) represents the dominant individual ligand–receptor interaction driving cytotoxic T-cell activation in this context.

The machine learning validation results—LR and Ridge—showed modest full-panel discrimination, and performance was only slightly above chance in BrighTNess. The G4 + G5 combination achieved the highest mean AUC of 0.653 from a post hoc screen but was not statistically superior to the full-panel models. The modest absolute AUC, while above chance in every fold, is consistent with the inherent biological heterogeneity of TNBC, the transcriptomic batch effects between independent RNA-seq platforms, and the imperfect alignment between a scRNA-seq prognosis-based discovery endpoint (Ki67 + lymphoid infiltrate) and a bulk pCR treatment response endpoint. The spatial transcriptomics validation—showing significantly higher G5 DNA damage and CD8 T-cell scores in Good Prognosis tissue spots and higher G4 Metabolism scores in Bad Prognosis tissue—provides independent tissue-level confirmation of the scRNA-seq findings, though the single-patient-per-group design of the available Visium data limits the statistical power of the co-localization analysis.

We acknowledge several limitations of this study. First, the scRNA-seq discovery cohort comprises only eight TNBC patients, which, while consistent with the largest publicly available TNBC single-cell atlas, limits the statistical power of the single-cell compositional findings and renders them hypothesis-generating rather than confirmatory. Second, the prognosis grouping is operationally defined by pathological lymphoid infiltration and clinical Ki67—not directly observed survival outcomes—and should not be interpreted as equivalent to clinical prognosis. Third, the bulk RNA-seq machine learning validation uses pathological complete response (pCR) as the endpoint, which, while a well-established surrogate for survival in TNBC, does not perfectly align with the lymphoid infiltration-based operational prognosis definition used in the scRNA-seq discovery phase; this endpoint misalignment may partially explain the modest cross-cohort AUC performance (mean AUC = 0.640–0.653). Fourth, CellChat-derived ligand–receptor interactions and scRNA-seq module scores represent computational inference from transcriptomic co-expression patterns and do not constitute experimental proof of direct cellular activation. Functional validation of the HLA → CD8 axis—for instance, using co-culture experiments with sorted G5 DNA-damage epithelial cells and autologous CD8 T cells—remains an important future direction to establish direct causality. Fifth, the spatial transcriptomics validation is restricted to one patient per prognosis group (n = 1 Good Prognosis: CID44971; n = 1 Bad Prognosis: CID4465), which severely limits statistical inference at the tissue level and precludes generalization of the spatial co-localization findings. These results are interpreted exclusively as hypothesis-generating spatial evidence consistent with the single-cell and bulk findings.

In conclusion, this study defines a mechanistic framework in which two opposing epithelial transcriptional states—G4 Metabolism and G5 DNA damage—determine the immune microenvironment landscape and chemotherapy response in TNBC. G5 DNA-damage epithelial cells, characterized by HORMAD1 expression and cGAS-STING pathway activation, upregulate MHC-I antigen presentation machinery and directly activate CD8 Cytotoxic T cells through HLA-A/B/C/E/F → CD8A/CD8B ligand–receptor interactions, defining the immune-active Good Prognosis state. Conversely, G4 Metabolism epithelial cells dominate Bad Prognosis tumors, driving immune exclusion and T-cell exhaustion through metabolic reprogramming. The G4 + G5 combination predicts pCR with consistent above-chance performance across three independent cohorts, providing a biologically interpretable prognostic framework. These findings support the rationale for combining DNA-damaging chemotherapy with PD-1/PD-L1 checkpoint blockade—where chemotherapy induces the G5 DNA-damage → MHC-I → CD8 activation axis, which is then sustained by checkpoint blockade—and suggest that cGAS-STING agonists may have therapeutic potential in Bad Prognosis TNBC patients whose tumors lack G5 epithelial cells and are therefore unable to mount a natural antigen-presentation response.

## Data Availability

The transcriptomic datasets analyzed in this study are publicly available through the Gene Expression Omnibus under accessions GSE176078, GSE163882, GSE164458, and GSE260693 and through the spatial-transcriptomics archive at Zenodo (https://doi.org/10.5281/zenodo.4739739, accessed on 1 June 2026). Derived numerical results are provided in Supplementary Tables S1–S17. All analysis code is available from the corresponding author upon reasonable request.

## Supplementary Materials

The following supporting information can be downloaded at: https://www.mdpi.com/article/10.3390/cimb48080846/s1.

## Abbreviations

The following abbreviations are used in this manuscript:

AUC	Area under the receiver operating characteristic curve
cGAS	Cyclic GMP–AMP synthase
GSEA	Gene-set enrichment analysis
HLA	Human leukocyte antigen
LOCO	Leave-one-cohort-out
MHC-I	Major histocompatibility complex class I
PCA	Principal component analysis
pCR	Pathological complete response
RD	Residual disease
RF	Random forest
scRNA-seq	Single-cell RNA sequencing
STING	Stimulator of interferon genes
TIL	Tumor-infiltrating lymphocyte
TME	Tumor immune microenvironment
TNBC	Triple-negative breast cancer
TPM	Transcripts per million
UMAP	Uniform manifold approximation and projection
