# Supplementary material for: Tumor-Intrinsic DNA Damage Signaling Is Associated with MHC-I Expression and CD8 Cytotoxic T-Cell Engagement in Triple-Negative Breast Cancer
Source: Curr Issues Mol Biol. 2026 Aug 20;48(8):846. doi: 10.3390/cimb48080846 (PMC13510773; doi:10.3390/cimb48080846)
Supplement: Supplementary file 1 [file cimb-48-00846-s001.zip › Supplementary Figures.pdf]

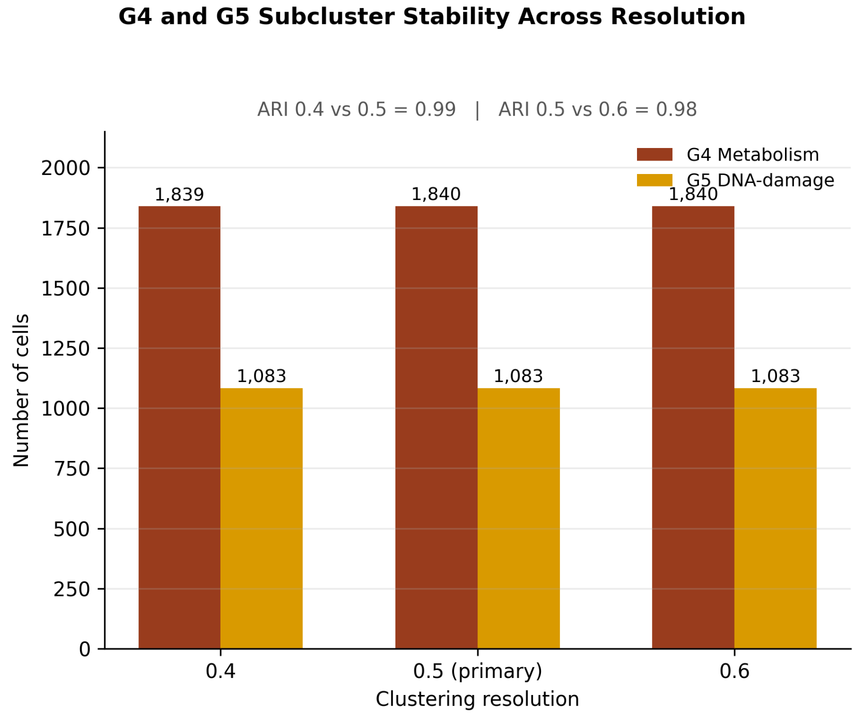

Supplementary Figure S1. Robustness of G4 Metabolism and G5 DNA-damage epithelial subclusters across clustering resolution. Cell counts per subcluster at resolutions 0.4, 0.5 (primary), and 0.6 are shown; subcluster composition remained essentially unchanged across this range (Adjusted Rand Index = 0.99 for resolution 0.4 vs. 0.5, and 0.98 for resolution 0.5 vs. 0.6).

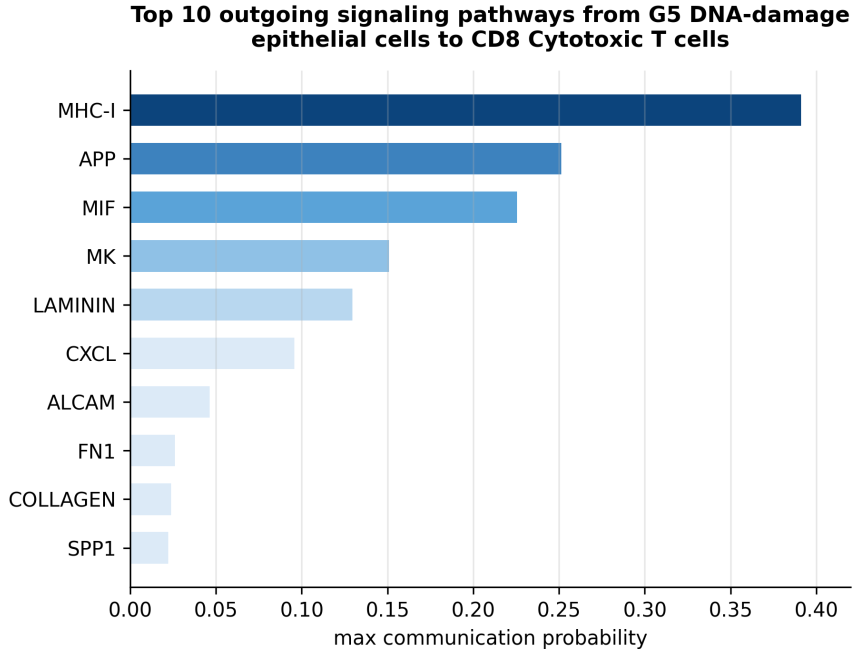

Supplementary Figure S2: Top 10 outgoing signaling pathways from G5 DNA-damage epithelial cells to CD8 Cytotoxic T cells, ranked by maximum communication probability (CellChat). MHC-I is the dominant pathway (max probability = 0.391), followed by APP (0.251) and MIF (0.226). Underlying data are provided in Supplementary Table S15.
